# Identification of microorganisms in persistent/secondary endodontic infections with respect to clinical and radiographic findings: bacterial culture and molecular detection

**Published:** 2019-04

**Authors:** Nazanin Zargar, Mahmoud Amin Marashi, Hengameh Ashraf, Rene Hakopian, Peyman Beigi

**Affiliations:** 1 Iranian Center for Endodontic Research, Research Institute of Dental Sciences, School of Dentistry, Shahid Beheshti University of Medical Sciences, Tehran, Iran; 2 Medical Microbiology Research Center, Qazvin University of Medical Sciences, Qazvin, Iran; 3 Department of Endodontics, School of Dentistry, Shahid Beheshti University of Medical Sciences, Tehran, Iran; 4 Department of Hematology, Faculty of Medicine, Tarbiat Modares University, Tehran, Iran

**Keywords:** Bacterial identification, Clinical signs, Polymerase chain reaction, Endodontic infection, 16S rDNA, *Enterococcus faecalis*

## Abstract

**Background and Objectives:**

Bacterial agents are commonly accepted as the main etiology of endodontic infections. A significant proportion of oral bacteria cannot be cultured using existing methods. Since diversity and abundance of bacterial species are different in different populations, the present study was aimed to identify effective microorganisms in persistent endodontic infections in Iranian patients based on culture and molecular biology methods using sequence analysis of 16S rDNA gene.

**Materials and Methods:**

Thirty patients with previous failure of endodontic treatment were enrolled in the study. After isolation and disinfection of the tooth surrounding area with 3% sodium hypochlorite and 30% hydrogen peroxide, sampling from the root canals was carried out using two sterile Hedstrom files and two sterile paper points, and then the specimens were transferred to the microbiology laboratory in thioglycolate transport medium so that they undergo aerobic-anaerobic culture, PCR, and 16S rDNA gene sequencing.

**Results:**

Of 30 patients (15 women and 15 men), 15 patients had radiographic lesions smaller than 5 mm and other 15 patients had radiographic lesions larger than 5 mm. The mean age of patients was 40.20 ± 13.76 years. A total of 26 patients were asymptomatic. Only four patients had clinical signs such as pain and percussion sensitivity and *Tannerella forsythia* was the most common bacterium found in this group of patients. 13 bacterial species were found in 11 different genus, one virus strain and one fungus strain. From 30 studied specimens, *Enterococcus faecalis* was the most common microorganism with prevalence rate of 63.63%.

**Conclusion:**

This study showed the type and prevalence of effective bacteria in secondary/persistent endodontic infections in Iranian patients. *E. faecalis* is the most commonly found microorganism in Iranian patients.

## INTRODUCTION

Bacterial agents are widely accepted as the main etiology of endodontic infections (1). Endodontic treatment failure often occurs due to the secondary/persistent endodontic infection (2–7). Persistent infection is usually characterized by the presence of an apical periodontitis or radiographic lesion. The disease, which occurs after root canal treatment failure, involves a large proportion of societies, even in advanced countries, and calculating the costs of root canal retreatments and dental restorations revealed dissipation of millions of dollars which imposed to the communities (8).

Inappropriate coronal seal, microleakage, failure in the chemical and mechanical preparation of the root canal, limitations and lack of proper quality of the canal filling material can result in a secondary or persistent endodontic infection that could cause apical periodontitis leading to failure of endodontic treatment (9).

Several microorganisms can cause endodontic treatment failure. *Enterococcus* species are prevalent in persistent infections, and *Enterococcus faecalis* has been found to be more common in these infections than other bacteria (2, 3, 7, 9–11). Fungi have also been found in primary endodontic infections, but their presence appears to be more prevalent in secondary/persistent endodontic infections and teeth with root canal treatment failure. *Candida albicans* is the most common prevalent fungus in the root canals of infected teeth (11–13). Recent studies have also proved the role of viruses such as *Cytomegalovirus* (CMV) and *Epstein–Barr virus* (EBV) in symptomatic periapical lesions (14, 15).

The major pathogens causing secondary endodontic infections can vary from country to country. One of the reasons for these differences is that various methods have been used for pathogens identification (9, 10, 16–19). Previously, microbial species and pathogens were identified using culture method, which was based on phenotypic and biochemical characteristics of microorganisms and associated with many limitations (20). Considering the wide-spread use of molecular biology techniques in recent years, 16S rDNA sequencing has played a major role in precise identification of isolated bacteria in modern microbiology. This method can not only diagnose the etiology of endodontic infections, but also gives clinicians, according to the existing microorganisms, the opportunity to determine the appropriate antimicrobial therapeutics, the duration of the treatment and the methods of infection control in the best way (2). Therefore, the current study was conducted to identify different bacterial species, some viruses, and one fungus strain in secondary or persitent endodontic infections with respect to clinical and radiographic findings in adult Iranian patients using culturing and the 16S rDNA sequencing methods.

## MATERIALS AND METHODS

This was an analytical cross-sectional study. The patients were selected from among referrals to the Endodontic Department of the School of Dentistry, Shahid Beheshti University of Medical Sciences. After ethics committee approval (IR.SBMU.IRDS. REC.1394.48), 30 patients with persistent root canal infection or previous root canal treatment failure were studied. Written informed consent was obtained from every patient before enrollment in the study. All of these patients had symptoms of endodontic infection after a clinical and radiographic examination. Persistent infection was assessed according to root canal filling quality (length and uniformity) and periapical lesion. These patients were placed in two groups of 15 patients based on the size of the periapical lesions in radiographs. The first group included patients having periapical lesions smaller than 5 mm in radiography and the second group with periapical lesions larger than 5 mm.

### Inclusion criteria.

1) Above 18 years of age, 2) lack of participation in another research project during the past 6 months, 3) teeth having problems for the use of rubber dam and isolation.

### Exclusion criteria.

1) Patient's lack of satisfaction to participate in the study, 2) Severe systemic diseases, 3) Pregnancy, 4) Use of any antibiotic in the last 30 days.

### Sampling method and implementation of research.

According to the previous studies, in total, 30 patients (15 women and 15 men) (2, 4) who referred to the School of Dentistry, Shahid Beheshti University of Medical Sciences met the inclusion criteria. All specimens were prepared in a completely similar manner by an endodontist. Hydrogen peroxide (30%) was used to disinfect the teeth and the surrounding area. This procedure was completed with 3% sodium hypochlorite. The restorations were removed and the access cavity to the pulp was prepared by a sterile high-speed carbide bur fully and rapidly. Then each tooth was isolated by rubberdam. Afterwards, the tooth and operatory field was disinfected again using 30% hydrogen peroxide followed by 3% sodium hypochlorite and finally neutralized by 5% sodium thiosulfate. The purpose behind repeating this action is to prevent the entry of microorganisms and debris from the access cavity into the root canal. This action was performed on the tooth surface as well as on the rubberdam. Sampling from the surrounding area was later performed to ensure the effectiveness of the isolation and the sterility of the area around the tooth and then investigated as with other specimen taken from the root canal (4, 7).

In the first stage, gutta-percha in the coronal part of the canal and the materials in the apical part of the canal were removed respectively using 2 and 3 Gates Glidden drills (Mani Inc Tokyo, Japan) and Hedstrom files (20 mm or 25 mm, Mani Inc Tokyo, Japan) in a sterile manner and without any chemicals. The contents of the canal were also transferred to the transport medium of thioglycolate (Merck, Darmstadt, Germany), if possible. A #20 Hedstrom file was inserted 1 mm shorter than the length of the canal, which was roughly measured by radiography, and removed after a brief filing operation. The cutting part of the file was separated from the file's handle with a sterile clipper and dropped into the same transport medium. The canal was then humidified by 9% normal saline and a #20 paper point (Dentsply-Maillefer, Ballaigues, Switzerland) was later inserted into the approximate length of the canal. The paper point was removed from the canal after one minute and transferred to the previous transport tube medium. In the next step, the shaving of the canal walls was performed using the #25 Hedstrom file. The cutting part of the file was separated and transferred to the same transport tube medium. Finally, the canal medium was wetted by normal saline and a #25 paper point was inserted into and remained in the canal for 1 minute. This paper point was also dropped in the same medium (10, 21).

### Laboratory steps.

The laboratory steps of this study were carried out in a chain fashion in two microbiology laboratories, School of Dentistry, Shahid Beheshti University of Medical Sciences and Microbiology Laboratory of Alborz University of Medical Sciences. Because different methods were used to identify cultivable and as yet uncultivable species, these species were separately investigated in this study.

### Cultivable species.

After completing the patient sampling process, the specimens were transferred to the microbiology laboratory in the transport medium. The extracted specimens contained in thioglycolate vial (Merck, Darmstadt, Germany) were cultured in 2 *Brucella* agar plates (Merck, Darmstadt, Germany) in laboratory under sterile conditions. There were two plates: one for aerobic species and the other one for anaerobic species. The aerobic culture plates were immediately incubated at 37°C. The anaerobic culture plates, along with a gas-pack (Merck, Darmstadt, Germany) were placed in the anaerobic jar and transferred to the incubator (Memmert, Schwabach, Germany). Both groups were kept in incubator for 48 to 72 hours in order to grow bacterial colonies. Isolation of bacteria was performed according to the morphologic characteristics of colonies and Gram staining. After primary detecting of each colony, based on its morphologic characteristics, a loop was isolated from each colony and used for DNA extraction using DNA extraction kit (Cinna Gen, Tehran, Iran).

### Uncultivable species.

A number of bacterial, viral and fungal species were pre-selected and their presence in the samples were separately examined (3, 10, 21). So, the tube content of each patient was directly investigated to identify the selected specific gene for each species primer. To complete the gene amplification step, species-specific primers were designed using AleIIID 6 software. The sequences of designed primers is shown in Table 1.

**Table 1.** PCR primer pairs used for detection of species/phylotypes in secondary endodontic infections

**Target**	**Primer pairs (5′-3′)**	**Lenght**	**Reference or source**
Actinobaculum oral clone EL030	AGA GTT TGA TCC TGG CTC AG CGC AGA ATC CGT GGA AAG A	(844)	(39)
*Atopobium parvulum*	AGA GTT TGA TCC TGG CTC AG TGC GGC ACG GAA GAA ATA CTC CCC		(39)
Synergistes oral clones BH017/D084	AGA GTT TGA TCC TGG CTC AG CGT CAA TGT TTC CAT CTC CTA C	(827)	(6)
Desulfobulbus oral clone R004	AGA GTT TGA TCC TGG CTC GAA GGC ACC ACC CAC TTT CAT GGG	(988)	(39)
Dialister invisus	CAG AAA TGC GGA GTT CTT CTT CG CCC GGG AAC GTA TTC ACC G	(1,035)	(6)
Synergistes oral clone BA121	AGA GTT TGA TCC TGG CTC AG TGC GAA AGG GTC GAT CCG C	(381)	(6)
Synergistes oral clone E3_33	AGA GTT TGA TCC TGG CTC AG ACA CTT GTA CGT CTC CAT ACA C	(999)	(6)
Olsenella profusa	AGA GTT TGA TCC TGG CTC AG TGC GGC ACG GAC GGA CAA TCC G	(1,000)	(6)
Olsenella uli	AGA GTT TGA TCC TGG CTC AG TGC GGC ACG GAG GGA TCG TCC C	(829)	(6)
TM7 oral clone I025	CCC TGC AGT GAG GGA TAA GA GTT TTC ATC GCT CGC TAA CTT G	(829)	(6)
*Candida albicans*	GCATCGATGAAGAACGCAGC	(477)	This study
HSV-1	TCCTCCGCTTATTGATATGC	(340)	This study
EBV	AGCTTGCGGGCCTCGTT	(115)	This study
Universal 16S rRNA gene	TACGTACAACCACATACAGC	(250)	(22)

### 16S rDNA sequencing.

After performing PCR and obtaining a band of about 1500 bp, we cut the PCR product with *Taq*I and *Hae*III enzymes to obtain proper patterns for sequencing. These patterns were assorted and one sample from every group was sent for sequencing. PCR was carried out for cultivable species using universal primers of 27F- (5′-AGA GTT TGA TCM TGG CTC AG-3′) and 1492R- (5′-CGG TTA CCT TGT TAC GAC TT-3′) (22). However, species-specific primers were used for non-cultivable species (Table 1). PCR materials and cycles were mentioned in Tables 2 and 3. Sequences were analyzed automatically in an ABI Prism 3100 Genetic Analyzer (Applied Biosystems –Hitachi, Japan).

**Table 2.** PCR reaction reagents composition

	**Material**	**Amount**
1	water	16 l
2	Mgcl_2_	0.5 μl
3	buffer	2.5 μl
4	dNTPs	0.3 μl
5	Primer (F, R)	0.5 μl
6	template	5 μl
7	TAQ	0.2 μl
8	Total	25 μl

**Table 3.** PCR program

	**Temperature**	**Time**	**Number of Cycles**
1	94°C	5 min	1
2	94°C	1 min	
3	61°C	1 min	30
4	72°C	2 min	
5	72°C	12 min	1
6	25°C	∞	1

Amplified products were separated by agarose (1%) gel electrophoresis in 0.5 × TBE, stained with ethidium bromide. The DNA was extracted from the bands on the gel using gel extraction kit (Qiagen, GmbH, Germany) and then was sequenced by MacrogenInc, Seoul, Korea.

### Statistical analysis.

Data were analyzed using SPSS software (V. 24; SPSS, Inc., Chicago, IL, United States). Frequency and percent of microorganism were reported in groups of PA lesions according to their sizes (<5 mm and >5 mm) and clinical symptoms. For statistical analysis Chi-square and Fisher exact tests were used. Comparisons were considered significant if P values were <0.05.

## RESULTS

Of 30 patients (15 women and 15 men), 15 had radiographic lesions smaller than 5 mm and 15 patients had radiographic lesions larger than 5 mm. The age of patients was between 23 and 70 years (mean age of 40.20 ± 13.76 years). 26 patients were asymptomatic (86.7%) and only their radiographic evidence was indicative of endodontic treatment failure. In this study 13 bacterial species belonging to 11 different genera, one strain of virus and one strain of fungi from the 30 studied samples were identified (Table 4). Based on the findings, *E. faecalis* and *Prevotella pallens* had the highest and lowest prevalence with prevalence rates of 63.33% and 6.66%, respectively. Other microorganisms are shown in Table 4 in terms of their prevalence rate.

**Table 4.** Frequency of detected microorganism in secondary endodontic infection according to the size of periapical lesions

**Species**	**Of teeth with PA lesions<5 mm (n=15)**	**Of teeth with PA lesions>5 mm (n=15)**	**P value**	**Total**
*Treponema denticola*	6 (40%)	6 (40%)	1.000	12 (40%)
*Streptococcus mitis*	5 (33.3%)	5 (33.3%)	1.000	10 (33.33%)
*Porphyromonas gingivalis*	3 (20%)	5 (33.3%)	0.682	8 (26.70%)
*Streptococcus salivarius*	7 (46.7%)	8 (53.3%)	0.715	15 (50%)
*Prevotella intermedia*	1 (6.7%)	5 (33.3%)	0.169	6 (20%)
*Tannerella forsythia*	7 (46.7%)	6 (40%)	0.713	13 (43.33%)
*Enterococcus faecalis*	12 (80%)	7 (46.7%)	0.058	19 (63.33%)
*Eikenella corrodens*	2 (13.3%)	2 (13.3%)	1.000	4 (13.33%)
*Treponema parvum*	3 (20%)	3 (20%)	1.000	6 (20%)
*Atopobium parvulum*	0 (0%)	3 (20%)	0.224	3 (10%)
*Dialister invisus*	8 (53.3%)	8 (53.3%)	1.000	16 (53.33%)
*Prevotella pallens*	0 (0%)	2 (13.3%)	0.483	2 (6.66%)
*Candida albicans*	6 (40%)	4 (26.7%)	0.439	10 (33.33%)
*HSV-1*	6 (40%)	4 (26.7%)	0.439	10 (33.33%)
*Fusobacterium nucleatum*	6 (40%)	4 (26.7%)	0.439	10 (33.33%)

In patients with periapical lesions less than 5 mm, *E. faecalis* was the most prevalent with 80% and *Dialister invisus* was placed in the second line with prevalence rate of 53.3%. However, in patients with periapical lesions greater than 5 mm, *Dialister invisus, Streptococcus salivarius* and *Treponema denticola* were the most prevalent (53.3%) and *E. faecalis* (46.7%) was in second place in terms of prevalence rate (Table 4).

Only 4 patients (3.3%) had clinical signs such as pain and percussion sensitivity and *Tannerella forsythia* was the most commonly found bacteria in this group of patients. Of these 4 patients 3 of them had periapical (PA) lesions larger than 5 mm and one of them had PA lesions smaller than 5 mm. The frequency of other microorganisms in relation to clinical symptoms is shown in Table 5.

**Table 5.** Frequency of detected microorganism in secondary endodontic infection according to the clinical findings

**Species**	**Number/percent of teeth without clinical symptoms**	**Number/percent of teeth with clinical symptoms**	**P value** 1.000
*Treponema denticola*	10 (38.5%)	2 (50%)	0.584
*Streptococcus mitis*	8 (30.8%)	2 (50%)	1.000
*Porphyromonas gingivalis*	7 (26.9%)	1 (25%)	0.598
*Streptococcus salivarius*	14 (53.8%)	1 (25%)	1.000
*Prevotella intermedia*	5 (19.2%)	1 (25%)	0.295
*Tannerella forsythia*	10 (38.5%)	3 (75%)	0.611
*Enterococcus faecalis*	17 (65.4%)	2 (50%)	0.454
*Eikenella corrodens*	3 (11.5%)	1 (25%)	0.557
*Treponema parvum*	6 (23.1%)	0 (0%)	1.000
*Atopobium parvulum*	3 (11.5%)	0 (0%)	1.000
*Dialister invisus*	14 (53.8%)	2 (50%)	1.000
*Prevotella pallens*	2 (7.7%)	0 (0%)	1.000
*Candida albicans*	9 (34.6%)	1 (25%)	0.272
*HSV-1*	10 (38.5%)	0 (0%)	0.584
*Fusobacterium nucleatum*	8 (30.8%)	2 (50%)	

Based on the findings of this study, *Atopobium parvulum* was found for the first time in the canals of teeth with persistent/secondary endodontic infections. Although the microorganisms and their frequencies were different in the groups of the study, however; according to the statistical analysis, the size of the periapical lesion had no effect on the frequency of detected microorganisms.

*Candida albicans* was seen in 10 cases (33.33%), with higher prevalence rate in the teeth with PA lesions smaller than 5mm (6/15 or 40%) in comparison with the teeth with larger lesions (4/15 or 26.7%). The prevalence of this fungus was also higher in asymptomatic teeth (34.6%) in comparison with symptomatic teeth (25%).

HSV 1 was more prevalent in teeth with PA lesions smaller than 5 mm (6/15 or 40%) than in teeth with larger PA lesions. It is also more prevalent in asymptomatic teeth (38.5%) than symptomatic teeth (0%).

## DISCUSSION

The microbial flora of the teeth with root canal treatment failure was previously identified for specific-species by culturing and PCR. In previous studies conducted in Japan, Germany, Scotland and the United States (2, 5, 23, 24), 16S rRNA gene sequencing was used as the microbial identification method. However, none of these studies used microbial culture and molecular method in parallel. This study is aimed to reduce the distance between the results of these two methods and to achieve accurate findings of the microbial composition of the persistent/secondary endodontic infection among the Iranian patients.

In this study, non-culturable species such as *Dialister invisus, Atopobium parvulum* and HSV-1 virus were found in specimens that prove the necessity to use molecular methods (25). However, the molecular method cannot accurately indicate the presence of all the microorganisms in the canal. Some microorganisms may exist in a very small number, so that their DNA cannot be detected by the 16S DNA sequencing method. A combination of the culture as well as culture-independent techniques would achieve a comprehensive microbial analysis of endodontic infections (5, 10).

A considerable number of bacteria can escape sampling with paper points disregarded root canal obturation materials so root canal filling sampling is of great importance in microbiological analysis and we did it (26). Also, due to presence of microorganisms in dentinal tubules, which could persist and resist after chemomechanical preparation of the canal walls, dentin shaving sample was taken by filing (21).

The results of sequencing of 16S rDNA gene in Iranian patients showed findings that were inconsistent with those obtained in other countries. In this study, *Atopobium parvulum* bacterium was discovered in persistent endodontic infections for the first time. The *Atopobium parvulum* is an uncultivable, Gram-positive and anaerobic bacterium belonging to *Actinobacteria* phylum, which was identified in 1993 for the first time (27). This bacterium had a prevalence of 10% in Iranian patients with a persistent / secondary endodontic infection, which has not been found in any other country, such as Germany, China, Japan, Brazil, South Korea and Sudan (5, 9, 16, 18, 19, 28). In a previous study, a bacterium of the same genus named *Atopobium rimae* was recovered from teeth with chronic apical abscesses only in Germany in 2015 (10).

Based on the results of our study, *E. faecalis* was the most prevalent microorganism (63.3%) in secondary endodontic infections in Iranian patients. *E. faecalis* can survive in endodontically treated teeth and has to resist intracanal disinfection procedures and intra canal medications such as calcium hydroxide (29). So this microorganism could have a role in endodontic treatment failures but the causation is unproved (30). This finding is consistent with the findings of studies in Germany (10), Brazil (7) and Japan (2), which indicates higher prevalence of this bacterium. However in some of the previous studies in other countries *E. faecalis* was not the predominant bacterium in secondary endodontic infections, even if present (3, 17). In this study the prevalence of *E. faecalis* in teeth with radiographic lesions smaller than 5 mm is higher. Kaufman found the higher prevalence of *E. faecalis* in endodontically treated teeth without periradicular lesions (31).

Another bacterium, which is the most prevalent in this study, was *Dialister invisus* (53.3%). This bacterium was detected in Germany with a lower prevalence (6).

The third most common bacterium in this study was *Streptococcus salivarius* with a prevalence of 50%. *Streptococcus* family bacteria was identified in studies conducted in China (16), Brazil (32) and Germany (6). It had an almost equal prevalence in the German population compared to the Iranian patients. The prevalence of *Streptococcus* in the root canals of teeth with / secondary/ persistent endodontic infections could be a reason for the resistance of these bacteria to endodontic treatments (32).

Comparison of 3 commonly identified bacteria in Iranian patients with the results of studies conducted in other countries indicates the difference between the dominant and the main bacteria causing secondary endodontic infection in different geographical areas (33). However, there are many similarities between the major bacteria found in Iran and Germany (6, 10). The differences in results may be due to a variation in sample sizes, studied specimens, studied populations, detection methods, and/or presence of some interventional factors. Other potential reasons for the debates may be attributed to the differences in the microbial communities of the various studied populations (34).

In this study, Herpes simplex virus 1 (HSV-1) was found in root canals of 10 patients, of which 6 patients had periapical lesions smaller than 5 mm and 4 patients with periapical lesions larger than 5 mm. This virus was formerly found in a study conducted in the United States with a lower prevalence rate (35). Although the HSV-1 virus was dominant in the Iranian patients, other studies showed Epstein–Barr Virus (EBV) was more prevalent (14, 15). In this study *Candida albicans* was found in 10 patients. This fungus was also identified in previous studies (6, 12), which can prove the role of fungi, and in particular *Candida albicans*, as a species resistant to common endodontic treatments.

In this study, 4 out of 30 patients had clinical symptoms such as pain or percussion sensitivity. *Tannerella forsythia* was the most common bacterium found in the symptomatic teeth. This finding was not consistent with other studies including the study conducted in Sudan (15), where the common bacteria were *firmicutes* and *fusobacteria*. This point may indicate that the spread of bacterial species in symptomatic lesions is greater than asymptomatic lesions. Also according to metagenomics studies it is possible to suggest a close relationship between several clinical features and greater microbiota diversity with persistent endodontic infection (36). It should be noted that the number of patients is different among these studies and the presence of only 4 symptomatic patients in our study is not enough to make any conclusion in this regard.

It is observed regarding the prevalence of bacteria that there is at least one Gram-positive bacterium in all specimens, such as *E. faecalis*, which can be a factor affecting the treatment failure.

In the root canal, fastidious microorganisms (eg, Spirochetes, or *Atopobium* spp.) may also be present. These microorganisms cannot be detected by culture methods. Therefore, specific DNA probes (PCR) have been used to detect unculturable microorganisms (37). Gene detection using PCR is a very sensitive method for bacterial identification (38). However lack of identification of a species in the study does not necessarily indicate the absence of that species in the target population and this technique is not the best molecular study. But because of the limitations of financial resources and accessibility to advanced techniques such as Next Generation Sequencing we used 16S rDNA sequencing.

## CONCLUSION

The findings of this study indicate that molecular biology methods and microbial culture methods are complementary to each other. In Iranian patients, *E. faecalis* (63.3%) is the most common microorganism in the root canal of the teeth with persistent / secondary infection. Based on the results, the type and prevalence of effective bacteria in persistent / secondary endodontic infections are different among the Iranian patients. Therefore, it is necessary to design the local and systemic treatment protocols in each country in accordance with the microbiota of that area.
